# Pathological transition as the arising mechanism for drug resistance in lung cancer

**DOI:** 10.1186/s40880-019-0402-8

**Published:** 2019-10-01

**Authors:** Yueqing Chen, Waiying Yvonne Tang, Xinyuan Tong, Hongbin Ji

**Affiliations:** 1 State Key Laboratory of Cell Biology, CAS Center for Excellence on Molecular Cell Science, Innovation Center for Cell Signaling Network, Institute of Biochemistry and Cell Biology, Shanghai Institutes for Biological Sciences, Chinese Academy of Sciences, Shanghai, 200031 P. R. China; 2 University of Chinese Academy of Sciences, Beijing, 100049 P. R. China; 3 Sunway Medical Centre, 47500 Bandar Sunway, Selangor Malaysia; 4 School of Life Science and Technology, Shanghai Tech University, Shanghai, 200120 P. R. China

**Keywords:** Lung cancer, Drug resistance, Pathological transition

## Abstract

Despite the tremendous efforts for improving therapeutics of lung cancer patients, its prognosis remains disappointing. This can be largely attributed to the lack of comprehensive understanding of drug resistance leading to insufficient development of effective therapeutics in clinic. Based on the current progresses of lung cancer research, we classify drug resistance mechanisms into three different levels: molecular, cellular and pathological level. All these three levels have significantly contributed to the acquisition and evolution of drug resistance in clinic. Our understanding on drug resistance mechanisms has begun to change the way of clinical practice and improve patient prognosis. In this review, we focus on discussing the pathological changes linking to drug resistance as this has been largely overlooked in the past decades.

## Background

Lung cancer can be classified into two main histological types: small cell lung cancer (SCLC) and non-small cell lung cancer (NSCLC). They account for approximately 15% and 85% of all lung cancers, respectively [1]. NSCLC can be further divided into three subtypes, namely lung adenocarcinoma (ADC), squamous cell carcinoma (SCC) and large cell carcinoma (LCC). These subtypes harbor different features, with distinct gene expression profiles [2] as well as lineage-specific biomarkers [3]. For example, lung ADC commonly express thyroid transcription factor-1 (TTF1, also known as NKX2-1) [4, 5], a p53-homologous nuclear protein mainly involved in basal cell commitment. LCC, as a pathologically heterogeneous entity which might represent solid ADC or non-keratinizing SCC, have no well-established biomarkers, yet [6]. In contrast to NSCLC, SCLC frequently express neuroendocrine markers, such as achaete-scute homologue 1 (ASCL1, also known as ASH1), neural cell adhesion molecule (NCAM) and synaptophysin (SYP) [7]. Lung ADC is frequently found at distal bronchioles [8], whereas SCC is often observed in more proximal airways [8]. Most lung ADC are considered as originating from alveolar type II (AT II) cells, club cells, or bronchial-alveolar stem cells (BASCs) [9], whereas SCC is observed at more proximal airway [6]. Most lung ADC are considered as originating from alveolar type II (AT II) cells, club cells, or bronchio-alveolar stem cells (BASCs) [7], whereas, lung SCC are mainly derived from basal cells located underneath trachea or bronchus epithelia [7]. SCLC arises from pulmonary neuro-endocrine (NE) cells and often spread along bronchi in a submucosal and circumferential fashion [8].

**Fig. 1:**
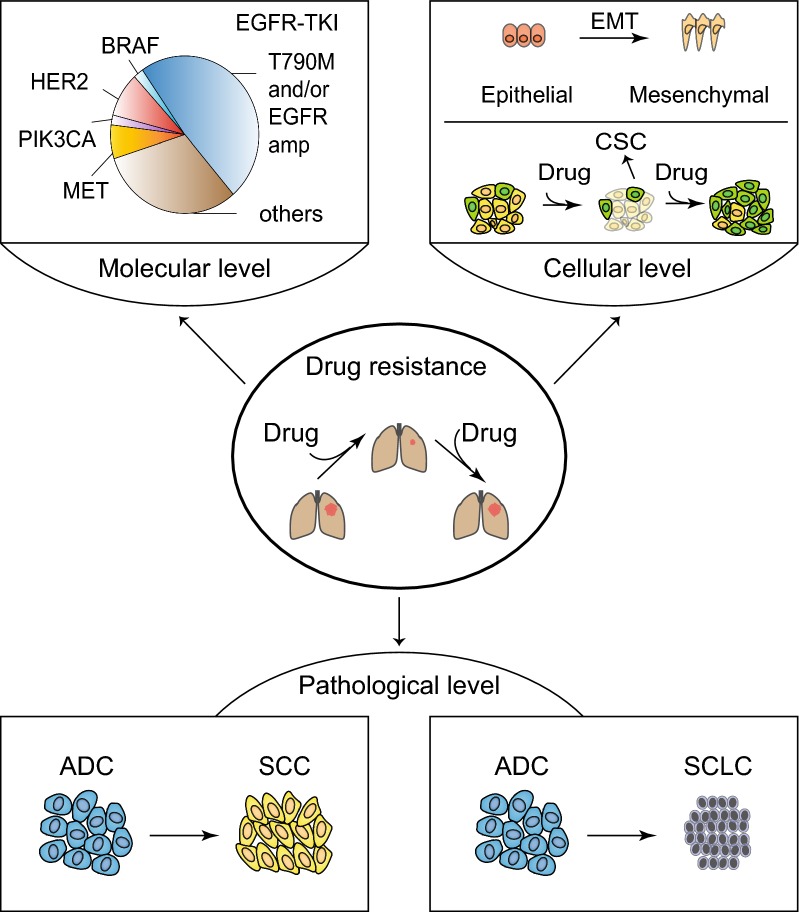
Three different levels of drug resistance mechanisms in lung cancer. Drug resistance develops at three different levels: molecular, cellular, and pathological level. Molecular level mechanism includes secondary EGFR T790M and MET amplification after the relapse from EGFR-TKI therapy. Cellular level mechanism mainly involves CSC and EMT. Pathological level mechanism includes the ADC-to-SCC transition and ADC or SCC-to-SCLC transition. *EGFR* epidermal growth factor receptor, *EGFR-TKI* epidermal growth factor receptor-tyrosine kinase inhibitor, *BRAF* serine/threonine-protein kinase B-raf, *HER2* receptor tyrosine-protein kinase erbB-2, *PIK3CA* phosphatidylinositol-4,5-bisphosphate 3-kinase catalytic subunit alpha, *MET* hepatocyte growth factor receptor, *EMT* epithelial-to-mesenchymal transition, *CSC* cell stem cell, *ADC* adenocarcinoma, *SCC* squamous cell carcinoma, *SCLC* small cell lung cancer

Despite of persistent medical efforts in last decades, lung cancer prognosis still remains disappointing, with a 5-year survival rate of approximately 15% [9]. This is in part attributed to the acquisition of early drug resistance. Understanding of drug resistance mechanisms hopefully improves therapeutic strategies and eventually changes clinical practice. We classify drug resistance mechanisms into three different levels: molecular, cellular and pathological level (Fig. 1). Although these three are closely linked with each other, changes in molecular level might occur in tumor initiation and development prior to those in the other two levels, which enable an early diagnosis with the usage of potential biomarkers. Previous studies have paid extensive attentions to the molecular and cellular level. In this review, we mainly focus on the pathological level which is largely unappreciated previously.

## Drug resistance mechanisms at molecular and cellular level

Molecular changes are frequently detected in relapsed patients after clinical treatment including chemotherapy, targeted therapy and immunotherapy. There are multiple drug resistance mechanisms at molecular level limit the effectiveness of chemotherapy, e.g., the deregulation of genes involved in drug uptake, cell cycle, apoptosis, sphingolipid metabolism as well as intracellular drug sequestration [10]. Gardner et al. [11] recently show that the down-regulation of Schlafen 11 (SLFN 11) mediated by enhancer of zeste 2 polycomb repressive complex 2 subunit (EZH2) and H3K27me3 modification induces DNA damage repair and thus enables SCLC chemo-resistance. Molecular alterations are also observed in relapsed patients after targeted therapy in lung cancers [12]. For example, during epidermal growth factor receptor-tyrosine kinase inhibitor (EGFR-TKI) treatment, secondary *EGFR* mutation T790M, MET proto-oncogene, receptor tyrosine kinase (*MET*, also known as hepatocyte growth factor receptor, *HGFR*) amplification, receptor tyrosine-protein kinase erbB-2 (*ERBB2*, also known as *HER2*) amplification as well as Kirsten rat sarcoma viral oncogene homolog (*KRAS)* mutations are often detectable in relapsed lung cancer patients and known to contribute to drug resistance [13–15]. In the case of anaplastic lymphoma kinase (*ALK*)-rearranged NSCLC patients, *ALK* point mutations, KIT proto-oncogene receptor tyrosine kinase amplification, and other driver mutations are implicated for disease relapse [13]. During the immune checkpoint blockade treatment, neo-antigen landscape shows dynamic change which contributes to the resistance to immunotherapy [16]. These data together support an important role of molecular alterations in orchestrating drug resistance.

Drug resistance mechanisms at cellular level is mainly classified into two types: cancer stem cell (CSC)- and epithelial-to-mesenchymal transition (EMT)-mediated drug resistance. CSCs are considered to be highly plastic, resistant to chemotherapy and capable to seed new aggressive and chemo-resistant tumors in distant organs [17]. Great efforts have been paid to investigate the vulnerability of CSC with the purpose to overcome drug resistance. However, the findings of reversible transition between CSC and non-CSC make the specific targeting of CSC extremely difficult [17]. The non-CSC, frequently as the major component of malignant tumors, is also known to harbor strong stemness and plasticity [18]. Such stemness and plasticity allows non-CSC to de-differentiate into CSC under stressful environment [18, 19]. Such de-differentiation together with the reversible transition between CSC and non-CSC creates a huge hurdle for effective targeting either CSC or non-CSC alone [19].

The transition from epithelial cells to mesenchymal cells also reflects the strong plasticity of cancer cells, which is frequently implicated in drug resistance. In contrast to epithelial cells, mesenchymal cells tend to harbor strong transforming growth factor beta (TGFβ) and Wnt autocrine signaling [20]. The EMT often associates with down-regulation of multiple apoptotic signaling pathways, while it enhances drug efflux and slows cell proliferation [17]. The EMT activates several processes including of programmed death-ligand 1 (PD-L1) expression elevation and tumor suppressor region 1 (TSP-1) secretion elevation, which induces immune suppression and promotes immune escape [17, 21]. Besides, several transcription factors including snail family transcriptional repressor (SNAIL), twist family bHLH transcription factor (TWIST) and zinc finger E-box binding homeobox 1 (ZEB1) activate classical EMT-associated properties and induce anti-apoptotic and pro-survival phenotype supporting malignant progression [22]. All these EMT-associated features collectively promote cancer cell survival and help them escape from effective drug treatment. However, this doesn’t always turn out to be true. A recent study shows that EMT triggered by EGFR-TKI treatment associates with decreased PD-L1 expression, indicative of the complexity of the link between EMT and immune checkpoint regulation [23].

## Drug resistance mechanisms at pathological level

**Fig. 2:**
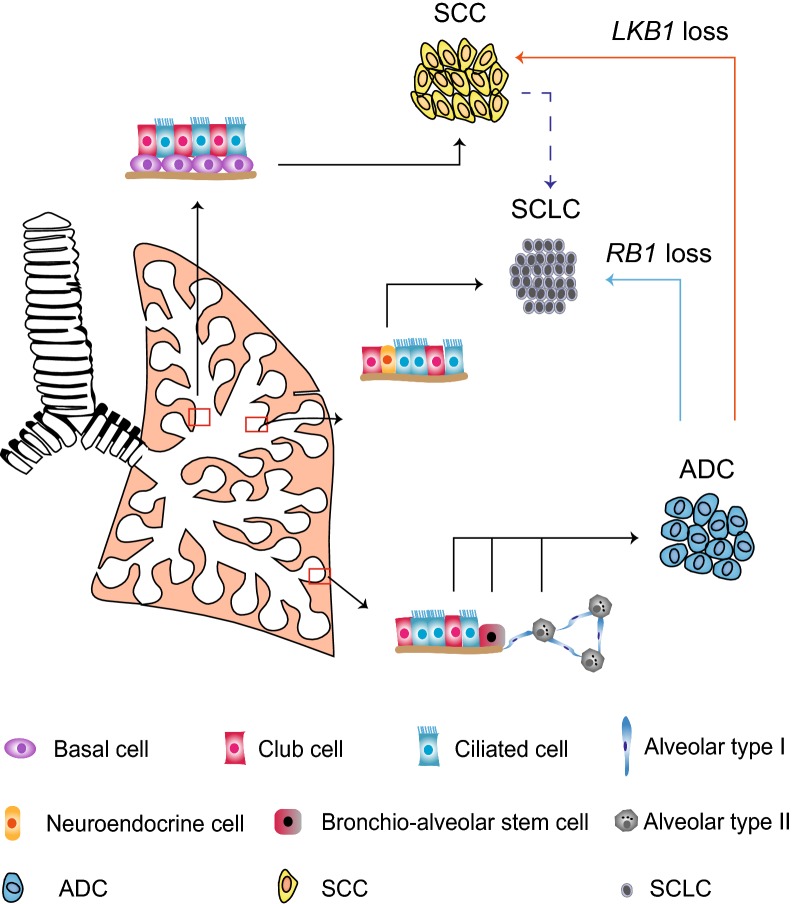
Pathological transition of different lung cancer subtypes. Lung cancer can be divided into two subtypes: NSCLC and SCLC. NSCLC can be further divided into three subtypes: ADC, SCC, and LCC. ADCs are considered to originate from alveolar type II cells, club cells or BASCs. SCC frequently found at more proximal airways is presumably derived from basal cells. SCLC is typically derived from neuroendocrine cells. Pathological transition is observed in clinic including lung ADC-to-SCC transdifferentiation and ADC or SCC-to-SCLC transition. Loss of *LKB1* or *RB1* potentially contributes to the squamous and SCLC transition, respectively. *NSCLC* non-small cell lung cancer, *SCLC* small cell lung cancer, *ADC* adenocarcinoma, *SCC* squamous cell carcinoma, *LCC* large cell carcinoma, *BASC* bronchio-alveolar stem cell, *LKB1* liver kinase B1, *RB1* retinoblastoma

Pathological transition has recently been implicated in clinic. Observation of lung ADC transition to either SCC or SCLC has been reported in relapsed patients [24, 25]. Below we summarized the current progress and discuss their link to drug resistance (Fig. 2).

## ADC/SCC-to-SCLC transition promotes drug resistance

Two large cohort studies reveal that approximately 5% human lung cancer display the mixed pathology such as adenosquamous carcinoma (Ad-SCC), combined large cell neuroendocrine carcinoma (LCNEC) and combined SCLC [26, 27]. Combined SCLC accounts for about 2.2% of all lung cancer [28]. Previous studies show that the SCLC and non-SCLC components of combined SCLC often share exactly the same genetic mutations [29, 30], indicating that these two different pathological lesions might share the same cells of origin and/or exist potential pathological transition. Notably, most of these pathologically mixed cancer are observed at advanced stages [26, 27], indicating that the potential pathological transition might occur during late stage of malignancy progression.

The SCLC transition is also observed in clinic after patient relapse from molecular targeted therapy. This is initially found in a woman with lung adenocarcinoma [31]. First biopsy shows that her tumor harbors EGFR exon 19 deletion and erlotinib treatment shows partial response. After 18 months of treatment, the tumor mass progresses. The second biopsy is then performed and shows SCLC pathology with the original EGFR exon 19 deletion, indicative of the potential transition from ADC to SCLC and its link to drug resistance. In later studies, researchers provided solid evidence showing that the transition of EGFR-mutant ADC to SCLC serves as a drug resistance mechanism. Sequist et al. [32] find that about 14% (5/37 cases) drug-resistant ADC cases transit to SCLC and thereby the standard SCLC therapy overcomes such resistance. We summarized the SCLC transition cases with available clinical details in Table 1. Among the total 33 cases, there are 14 males and 19 females. Despite of those unknown smoking status, about 77% (20/26) of the patients are non-smokers. There seems no preference for gender and smoking status. Except for 8 cases with unknown mutation status, most patients (96%, 24/25) show exactly the same oncogenic EGFR mutations or ALK fusions in the first and second biopsies. Additional mutations such as phosphatidylinositol-4,5-bisphosphate 3-kinase catalytic subunit alpha (PIK3CA) mutation, EGFR T790M are also detectable after relapse in 1 and 2 cases, respectively, indicative of the combined mechanisms at both molecular and pathological levels [13].

**Table 1:** Characteristics of 33 relapsed lung ADC patients with SCLC transition

Patient ID	Gender	Age	Smoking status	Therapy	1st biopsy^a^		2nd biopsy^b^		References
					Pathological status	Mutation status	Pathological status	Mutation status	
1	M	54	NA	TKI	ADC	EGFR 19 del	SCLC	EGFR 19 del	[32]
2	F	56	NA	TKI	ADC	EGFR 19 del	SCLC	EGFR 19 del	[32]
3	F	61	NA	TKI	ADC	EGFR 19 del	SCLC	EGFR 19 del	[32]
4	F	72	N	Gef	ADC	EGFR 19 del	SCLC	EGFR 19 del	[91]
5	F	46	N	Erl	ADC	EGFR 19 del	SCLC	EGFR 19 del	[92]
6	F	52	N	Erl	ADC	EGFR 19 del	SCLC	EGFR 19 del	[93]
7	M	80	N	Ico	ADC	EGFR 19 del	SCLC	EGFR 19 del	[94]
8	F	63	N	Erl	ADC	EGFR 19 del	SCLC	EGFR 19 del	[95]
9	M	46	Y	Gef	ADC	EGFR 19 del	SCLC	EGFR 19 del	[96]
10	M	49	Y	Erl	ADC	EGFR 19 del and FGFR3 exon 17 deletion	SCLC	EGFR 19 del and FGFR3 exon 17 deletion	[97]
11	F	60	N	Gef	ADC	EGFR 19 del	SCLC	EGFR 19 del	[98]
12	M	65	N	Afa	ADC	EGFR 19 del	SCLC	EGFR 19 del	[99]
13	F	37	N	Gef	ADC	EGFR 19 del	SCLC	EGFR 19 del + T790M	[100]
14	F	42	N	Erl	ADC	EGFR 19 del	SCLC	EGFR 19 del + T790M	[101]
15	F	49	N	Gef	ADC	EGFR 19 del	SCLC	NA	[102]
16	M	41	Y	Gef	ADC	EGFR 19 del	SCLC + SCC	NA	[61]
17	M	74	Y	Gef	ADC	EGFR 19 del	SCLC	WT	[103]
18	F	48	NA	TKI	ADC	EGFR L858R	SCLC	EGFR L858R	[32]
19	F	67	NA	TKI	ADC	EGFR L858R	SCLC	EGFR L858R	[32]
20	F	72	N	Gef	ADC	EGFR L858R	SCLC	EGFR L858R	[104]
21	M	46	N	Gef	ADC	EGFR L858R	SCLC	EGFR L858R	[105]
22	M	49	Y	Gef	ADC	EGFR L858R	SCLC	EGFR L858R	[106]
23	F	65	N	Gef	ADC	EGFR L858R	SCLC	EGFR L858R	[107]
24	M	73	NA	Gef	ADC	EGFR L858R	SCLC	EGFR L858R	[108]
25	F	40	NA	TKI	ADC	EGFR L858R	SCLC	EGFR L858R and PIK3CA	[32]
26	M	38	N	Erl	ADC	EGFR L858R	SCLC	NA	[109]
27	M	72	Y	Crizo	ADC	ALK	SCLC	ALK	[33]
28	M	67	N	Alec	ADC	ALK	SCLC	ALK	[34]
29	F	72	N	Gef	ADC	WT	SCLC	NA	[35]
30	M	61	N	TKI	ADC	NA	SCLC	EGFR 19 del	[110]
31	F	46	N	Gef	ADC	NA	SCLC	EGFR 19 del	[111]
32	F	45	N	Erl- Gef	ADC	NA	SCLC	EGFR 19 del	[31]
33	F	73	N	Gef	ADC	NA	SCLC	EGFR L858R	[104]

*Y* yes, *N* no, *NA* not available, *M* male, *F* female, *ADC* adenocarcinoma, *SCC* squamous cell carcinoma, *SCLC* small cell lung cancer, *TKI* tyrosine kinase inhibitor, *Gef* gefitinib, *Erl* erlotinib, *Ico* icotinib, *Afa* afatinib, *Crizo* crizotinib, *Alec* alectinib, *EGFR* epidermal growth factor receptor, *EGFR 19 del* EGFR exon 19 deletion, *ALK* anaplastic lymphoma kinase, *WT* wild-type

^a^1st biopsy: the first biopsy

^b^2nd biopsy: the second biopsy

Transition from ADC to SCLC is also observed in patients with ALK rearrangement. Two relapsed patients with ALK fusion after receiving alectinib or crizotinib treatment showed ADC-to-SCLC transition [33, 34]. Another patient with wild-type EGFR also showed the transition to SCLC after developing TKI resistance [35]. These data indicate that the transition to SCLC might be independent of various oncogenic drivers.

The transition from SCC to SCLC is also associated with drug resistance in the clinic [28]. After receiving surgery or radiation and chemotherapy, a total of 16 SCC patients were found to have SCLC transition. The majority of transited SCLC (12/16, 75%) is found to locate at the same sites as the primary tumors.

Recent studies have begun to uncover the underlying mechanisms involved in the ADC-to-SCLC transition [25, 36]. Niederst et al. [25] found that retinoblastoma (*RB*) was universally lost in all transited SCLC. This is consistent with previous finding about the concurrent loss of *RB* and *p53* alleles in most SCLC [37]. Consistently, Owen et al. [38] find that both RB and P53 deficiencies are required to reprogram lung epithelial cells to SCLC. Despite of the genomic evidence of *EGFR* mutations in transited SCLC, the expression of *EGFR* mutants are found to be remarkably decreased or even shut off [25]. Whether *RB* loss contributes to such down-regulation of *EGFR* level remains unknown. However, the decreased EGFR expression provides a reasonable explanation for the TKI resistance in transited SCLC [36].

## Lung ADC-to-SCC transition links to drug resistance

Lung Ad-SCC is the major subtype of pathologically mixed lung cancer. Ad-SCC contains both adenomatous and squamous pathology [39] and accounts for approximately 60%–75% of all mixed lung cancer [26, 27]. Similar to combined SCLC, the adenomatous and squamous components in Ad-SCC frequently share the same genetic alterations [40–43], indicative of potential pathological transition. Up to date, about 22 reported cases support the link between the ADC-to-SCC transition (AST) and drug resistance (Table 2). Among these patients, the majority (81.8%) is female and 12 (66.7%) of them are non-smoker. Almost all of the transited SCC displays the same *EGFR* mutations as detected in ADC. *EGFR* T790M and *PIK3CA* mutations were also detected in 4 (18.2%) patients, indicative of complicate resistance mechanisms. Moreover, 2 *ALK*-fusion patients showed AST after the relapse from molecular targeted therapy. AST were also detected in two patients with wild-type EGFR. Except for molecular targeted therapy, AST was also found in patients treated with chemotherapy or immunotherapy. Two patients received chemotherapy and one received chemotherapy and immunotherapy were found to have SCC transition at relapse. These data convincingly support the important link between AST and drug resistance.

**Table 2:** Characteristics of 22 relapsed lung ADC patients with potential squamous transition

Patient ID	Gender	Age	Smoking status	Therapy	1st biopsy^a^		2nd biopsy^b^		References
					Pathological status	Mutation status	Pathological status	Mutation status	
1	F	79	N	Chemotherapy	ADC	EGFR 19 del	SCC	EGFR 19 del	[112]
2	M	43	Y	Chemotherapy	ADC	EGFR 19 del	SCC	EGFR 19 del	[113]
3	F	48	N	Gef	ADC	EGFR 19 del	SCC	EGFR 19 del	[114]
4	F	51	NA	Gef	ADC	EGFR 19 del	SCC	EGFR 19 del	[115]
5	F	58	Y	Erl	ADC	EGFR 19 del	SCC	EGFR 19 del	[116]
6	F	66	N	Erl	ADC	EGFR 19 del	SCC	EGFR 19 del	[117]
7	F	67	NA	Afa	ADC	EGFR 19 del	SCC	EGFR 19 del and PIK3CA mutation	[118]
8	F	40	Y	Afa	ADC	EGFR 19 del	SCC	EGFR 19 del + T790M	[119]
9	F	79	N	Gef	ADC	EGFR 19 del	SCC	EGFR L858R + T790M	[120]
10	M	41	Y	Gef	ADC	EGFR 19 del	SCC + SCLC	NA	[61]
11	F	52	Y	Erl + Beva	ADC	EGFR 19 del	SCC	EGFR 19 del	[121]
12	F	61	N	Gef	ADC	EGFR L858R	SCC	EGFR L858R	[115]
13	M	62	N	Gef	ADC	EGFR L858R	SCC	EGFR L858R	[122]
14	F	63	N	Erl	ADC	EGFR L858R	SCC	EGFR L858R and PIK3CA	[123]
15	F	74	Y	Gef	ADC	EGFR L858R	SCC	EGFR L858R + T790M	[120]
16	M	68	Y	Erl	ADC	EGFR L858R	SCC	EGFR L858R + T790M	[124]
17	F	43	Y	Gef	ADC	EGFR L858R	SCC	EGFR L858R +S768I	[125]
18	F	64	N	Gef	ADC	EGFR L858R + T790M	SCC	EGFR L858R + T790M	[114]
19	F	60	Y	ALK TKI	ADC	ALK	SCC	ALK	[126]
20	F	52	N	Crizo/Alec	ADC	ALK	SCC	ALK	[127]
21	F	63	N	Erl	ADC	WT	SCC	EGFR L858R + T790M	[128]
22	M	69	N	Chemotherapy–immunotherapy	ADC	WT	SCC	NA	[129]

*Y* yes, *N* no, *NA* not available, *M* male, *F* female, *ADC* adenocarcinoma, *SCC* squamous cell carcinoma, *SCLC* small cell lung cancer, *EGFR* epidermal growth factor receptor, *TKI* tyrosine kinase inhibitor, *Gef* gefitinib, *Erl* erlotinib, *Afa* afatinib, *Crizo* crizotinib, *Alec* alectinib, *Ceri* ceritinib, *Beva* bevacizumab, *ALK* anaplastic lymphoma kinase, *WT* wild type, *EGFR 19 del* EGFR exon 19 deletion

^a^1st biopsy: the first biopsy

^b^2nd biopsy: the second biopsy

## Evidence from animal models supporting the ADC-to-SCC transition

Studies of the Genetically Engineered Mouse Models (GEMMs) have provided strong in vivo evidence in supporting the ADC-to-SCC transition [44, 45]. We and others have previously found that liver kinase B1 (*LKB1*, also named as *STK11*) is frequently mutated in human lung ADC, SCC as well as Ad-SCC [46, 47]. Inactivating mutations of *LKB1* seem to be significantly concurrent with *Kras* mutations and confers lung ADC with strong malignant potential and promotes metastasis [46, 48]. Strikingly, *Lkb1* deletion in *Kras*^G12D^ GEMMs could make ADC progressively transition into SCC via metabolic reprogramming and excessive accumulation of reactive oxygen species (ROS) [49]. YAP, the major transcriptional co-factor of the Hippo pathway, functions as the barrier for AST. When *Lkb1* is lost in lung ADC, YAP is activated and up-regulates ZEB2 expression, which in turn represses DNp63 transcription. During the malignant progression and when ADC grows big, the deficiency of extracellular matrix (ECM), e.g., decreased collagen deposition, fails to promote YAP activation and thus relieves ZEB2-mediated repression of DNp63 expression and eventually triggers the AST program [50].

The lysyl oxidase (LOX) family are responsible for cross-linking collagen and elastin, and thus importantly maintain the rigidity and structural stability of ECM [51]. The LOX family has five members including LOX, LOXL1, LOXL2, LOXL3 and LOXL4 with similar catalytic activities [51]. Previous study shows that LOX importantly regulates AST through ECM remodeling [44, 49]. During the AST process, LOX decreases with concurrent reduction of collagen disposition [44]. Pharmacological inhibition of LOX significantly accelerates the AST process in *Kras*^G12D^/*Lkb1*^L/L^ (*KL)* model [44]. More importantly, long-term LOX inhibition could trigger AST even in *Kras*^G12D^/*Trp53*^L/L^ (*KP*) mouse model, which is known to produce lung ADC only [52]. This highlights an essential role of LOX and ECM remodeling in AST, which is independent of *LKB1* deficiency [52]. The transited SCC show strong resistance to LOX inhibition in contrast to lung ADC, consistent with the association of AST and drug resistance [52].

Chromatin analysis reveals the contribution of epigenetic regulation to AST process in *KL* model. The transited SCC are featured with the decrease of H3K27me3 level and the increase of H3K27ac and H3K4me3 levels, which might be involved in regulating several key squamous-associated genes such as *Sox2*, *∆Np63* and *Ngfr* [45]. EZH2, the methyltransferase responsible for catalyzing H3K27me3 [53], is highly expressed in transited SCC. Similar findings are observed in human lung SCC and the squamous component of human Ad-SCC [45].

Interestingly, *Lkb1* loss together with ectopic SOX2 expression promotes the development of SCC, potentially through the progressive transition from ADC to SCC [54]. Simultaneous deletion of *FoxA1*/*2* and *Nkx2*-*1* in KRAS mouse model promotes the transition from ADC to SCC and these tumors are somehow different from those in KL model, and featured with keratinizing squamous cell carcinomas [55]. It remains interesting to see whether LKB1 is inactivated in this model and the squamous transition links to drug resistance.

Previous work has demonstrated that BASCs and club cells are the main cell types for squamous transition [45]. Up to date, most techniques used to isolate BASCs are based on FACS sorting. We recently take advantage of dual recombinant systems including Cre/LoxP and Dre/Rox systems to do the specific lineage-tracing of BASCs in vivo [56]. We found that BASCs are capable of differentiating into multiple cell lineages including club cells, ciliated cells, alveolar type I and type II cells in various lung injury models [56]. Future work will be interesting to illustrate whether BASC-derived tumors are prone to transdifferentiate into SCC when *Lkb1* is deleted.

## Conclusion

The drug resistance mechanisms can be classified into three different levels: molecular, cellular and pathological level. The ADC-to-SCLC transition and ADC-to-SCC transition are two major patterns for pathological transition in link to acquired drug resistance. A better understanding of drug resistance mechanisms will hopefully change the way of clinical practice and improve patient prognosis.

## Perspectives

It’s well established that the pathology of lung cancer serves as an important factor for clinical management, e.g., lung ADC, SCC and SCLC are therapeutically treated differently. The link between pathological transition and drug resistance indicates that even in the era of targeted therapy, the importance of pathology should not be neglected. For example, in *EGFR*-mutant lung ADC, the median progression-free survival (PFS) after TKI treatment is about 10–13 months [57]. In contrast, the PFS for lung SCC with similar EGFR mutations is only 2.4 months [57]. Even more dramatic finding is that transited SCLC from *EGFR*-mutant ADC have almost no response to TKI, potentially due to the shut-down of EGFR transcription [36]. This could be explained by the ‘missing target’ theory, in which the therapeutic target disappears after long-term treatment and drug resistance development. It will be interesting to test how *EGFR* mutants are epigenetically regulated and how we could transcriptionally re-activate *EGFR* mutants, which might help develop novel therapeutic strategies to overcome drug resistance in these transited SCLC.

Recent studies have also indicated that therapeutic targets could “face off” between different pathologies, e.g., *YAP* works as proto-oncogene in lung ADC but tumor suppressor in SCC. In human lung ADC, YAP is highly expressed and associated with poor prognosis [58]. Consistently, ectopic YAP expression accelerates lung ADC progression in *Kras*^*G12D*^ mouse model [59]. In contrast, YAP suppresses lung SCC progression potentially through down-regulation of the lineage-survival gene DNP63 [60]. We find that digitoxin is highly potent in suppressing SCC growth through YAP activation [60]. Understanding of the double faces of YAP in lung ADC and SCC will certainly help gain novel insights into the process of AST and pathological transition.

Interestingly, pathological transition is not limited to AST or ADC/SCC-to-SCLC transition. Although rare, one case report shows the transition of *EGFR*-mutated ADC to both SCC and SCLC [61]. Pathological transition is also observed when comparing the primary tumor with brain metastases [62]. For example, two patients with primary lung ADC show SCC or SCLC in brain metastasis whereas another patient with primary lung SCC shows ADC in brain metastasis. Transitions from ADC to LCNEC, or Ad-SCC to SCLC have also been reported [63, 64]. Obviously, our understanding of pathological transition is still very limited. Most case reports are from the *EGFR*-mutant lung cancer patients who are ethically treated with multiple biopsies. With the improvement of treatment strategies, multiple biopsies specimens will hopefully provide more insightful information about pathological transition.

**Fig. 3:**
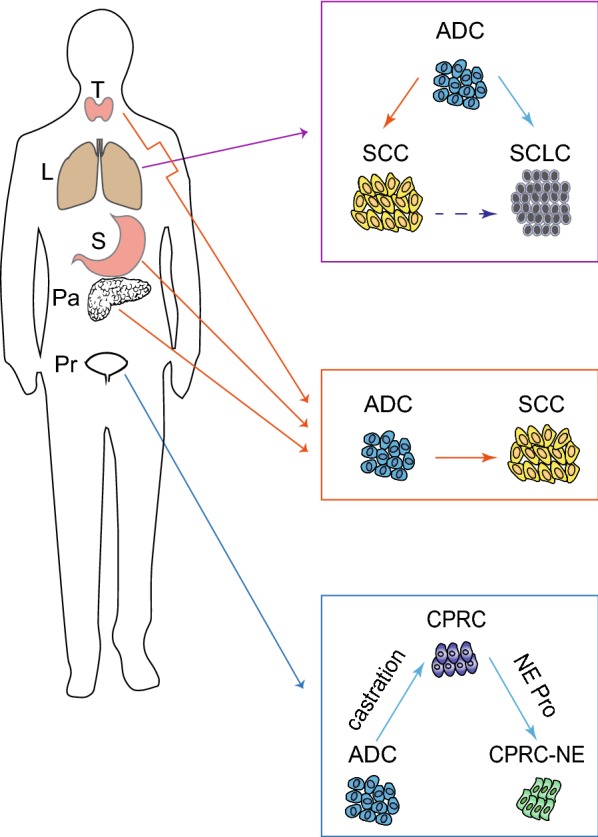
Pathological transition of different types of cancers. Pathological transition in lung cancer includes AST and ADC or SCC-to-SCLC transition. The AST or AST-like process is previously reported in thyroid gland carcinoma, pancreatic cancer as well as gastric cancer. Moreover, neuroendocrine differentiation in ADC has also been reported in prostate cancer. *T* thyroid gland, *L* lung, *S* stomach, *Pa* pancreas, *Pr* prostate, *Pro* proliferation, *SCLC* small cell lung cancer, *ADC* adenocarcinoma, *SCC* squamous cell carcinoma, *AST* ADC to SCC transition, *CPRC* castration-resistant prostate cancer, *NE* neuroendocrine

Besides lung cancer, mixed pathologies are also observed in other types of cancer (Fig. 3), e.g., Ad-SCC are detectable in human colon cancer [65], prostate cancer [66–76] and pancreatic cancer [77]. The AST or AST-like process has been previously reported in pancreatic cancer [78], thyroid gland carcinoma [79–81] as well as gastric cancer [82]. Moreover, neuroendocrine differentiation in ADC has been reported in prostate cancer. After androgen receptor (AR) treatment in castration-resistant prostate cancer (CPRC), certain patients develop neuroendocrine small cell cancer (CRPC-NE) [83–85]. It is understandable that the malignant transformation of benign tumors induce drug resistance [86]. Interestingly, malignant tumors may sometimes become less aggressive or even benign after chemotherapy, such as neuroblastoma to ganglioneuroma transition [87] and malignant germ cell tumors to teratoma transition [88–90]. These transformed tumors are still growing but show the resistance to chemotherapy.

Together, these findings suggest that pathological transition might be more common than we previously thought. No doubt, the better understanding of pathological transition and the link with drug resistance will be beneficial for future clinic practice and eventually help cancer patients.

## Data Availability

The datasets generated and/or analyzed during the current study are available in the Pubmed repository. For Table 1, we used one or a combination of the following terms in PubMed and selected articles on the basis of relevance to transition from ADC to SCLC: ‘non-small cell lung cancer’, ‘transition’, ‘transformation’, ‘transdifferentiation’, ‘adenocarcinoma’, ‘small cell carcinoma’ and ‘small cell lung cancer’. All dates and languages are included in the search. We only collect case reports with detailed clinical information. For Table 2, we used one or a combination of the following terms in PubMed and selected articles on the basis of relevance to transformation from ADC to SCC: ‘transition’, ‘transformation’, ‘transdifferentiation’, ‘adenocarcinoma’ and ‘squamous cell carcinoma’. All dates and languages are included in the search. We only collect case reports with detailed clinical information.

## Abbreviations

SCLC: small cell lung cancer
NSCLC: non-small cell lung cancer
ADC: adenocarcinoma
SCC: squamous cell carcinoma
LCC: large cell carcinoma
TTF1: thyroid transcription factor-1
DNP63: delta N tumor protein 63
ASCL1: achaete-scute homologue 1
NCAM: neural cell adhesion molecule
SYP: synaptophysin
AT II: alveolar type II
BASC: bronchio-alveolar stem cell
NE: neuro-endocrine
SLFN 11: schlafen 11
EZH2: enhancer of zeste 2 polycomb repressive complex 2 subunit
EGFR: epidermal growth factor receptor
MET: MET proto-oncogene, receptor tyrosine kinase (also known as HGFR)
HGFR: hepatocyte growth factor receptor
ERBB2: receptor tyrosine-protein kinase erbB-2 (also known as HER2)
KRAS: kirsten rat sarcoma viral oncogene homolog
ALK: anaplastic lymphoma kinase
KIT: KIT proto-oncogene receptor tyrosine kinase
TGFβ: transforming growth factor beta
EMT: epithelial-to-mesenchymal transition
PD-L1: programmed death-ligand 1
TSP-1: tumor suppressor region 1
SNAIL: snail family transcriptional repressor
TWIST: wist family bHLH transcription factor
ZEB1: zinc finger E-box binding homeobox 1
Ad-SCC: adenosquamous carcinoma
LCNEC: large cell neuroendocrine carcinoma
RB: retinoblastoma
AST: ADC-to-SCC transition
GEMM: Genetically Engineered Mouse Model
LKB1: liver kinase B1
ROS: reactive oxygen species
ECM: extracellular matrix
LOX: lysyl oxidase
KL: Kras^G12D^/Lkb1^L/L^
KP: Kras^G12D^/Trp53^L/L^
PFS: progression-free survival
EGFR-TKI: EGFR-tyrosine kinase inhibitor
CSC: cancer stem cell
AR: androgen receptor
CPRC: castration-resistant prostate cancer
CRPC-NE: neuroendocrine small cell cancer
